# Clinicopathological Significance of ATRX Expression in Nasopharyngeal Carcinoma Patients: A Retrospective Study

**DOI:** 10.7150/jca.63333

**Published:** 2021-10-03

**Authors:** Yangchun Xie, Haihua Wang, Sisi Wang, Yuhua Feng, Yeqian Feng, Songqing Fan, Chunhong Hu, Xianling Liu, Tao Hou

**Affiliations:** 1 Department of Oncology, The Second Xiangya Hospital, Central South University, Changsha, Hunan, 410011, China.; 2 Department of Pathology, The Second Xiangya Hospital, Central South University, Changsha, Hunan, 410011, China.

**Keywords:** Nasopharyngeal Carcinoma, ATRX, Staging, Survival, Prognosis

## Abstract

**Background:** Nasopharyngeal carcinoma (NPC) is the most common head and neck squamous cell carcinoma in south China. Radiation technology improves the local control rates in early NPC. However, the distant metastases are still the main cause of treatment failure. Thus, to find biomarkers for prognosis will help to enhance the survival of NPC. ATRX is a chromatin remodeling protein localized in the nucleus. Deletion or mutation of ATRX gene has been demonstrated in a variety of malignancies. However, the significance of ATRX expression in the prognosis of NPC remains unclear.

**Methods:** Tumor tissues from 227 NPC patients diagnosed in the Second Xiangya Hospital of Central South University from 2011 to 2016 were selected. Immunohistochemistry was used to detect the ATRX expression level of the tumor tissue. Chi-square test was used to analyze the relationship between ATRX expression and clinical characteristics such as age, sex, T stage, N stage and clinical stage. Kaplan-Meier method was used for survival analysis, and log-rank was used to compare the difference in survival rate.

**Results:** There were 53 patients with negative ATRX expression, accounting for 24.2% of the total group. ATRX expression was not significantly associated with age, sex, N stage, clinical stage, and progression-free survival (PFS) (P>0.05). However, patients with negative ATRX expression had earlier T staging (P=0.045) and a higher 5-year overall survival (84.9% vs 66.9%, P=0.022).

**Conclusions:** Loss of ATRX expression may contribute to better prognosis in patients with NPC.

## Introduction

Nasopharyngeal Carcinoma (NPC) is the most common head and neck squamous cell carcinoma in south China and southeastern Asia 1, 2. Due to its radiosensitive behavior, radiation (RT) and chemoradiotherapy (CRT) has become the cornerstone of the treatment for early or locoregionally advanced diseases 3, 4. However, about 25-30% of the patients will suffer from disease relapse or metastasis, and the prognosis of advanced patients is poor. The biological behavior of NPC varied dramatically in individual patients. Therefore, seeking for biomarkers for survival prediction, patient stratification and treatment adaptation is urgently needed 5.

Alpha thalassemia/mental retardation X-linked (ATRX) is a protein containing an ATPase/helicase domain, which belongs to the switch/sucrose nonfermentable (SWI/SNF) family of chromatin remodeling proteins 6. It is found to be involved in cell cycle-dependent phosphorylation, which regulates its nuclear matrix and chromatin association and suggests its involvement in gene regulation at interphase and chromosomal segregation in mitosis 7. It has been demonstrated that ATRX is frequently mutated in a variety of mesenchymal tumors including gliomas 8, neuroendocrine neoplasms 9, and sarcomas 10. ATRX mutation leads to decreased ATRX protein expression, and results in tumor genome instability, higher tumor mutation burden, and thus leading to increased sensitivity to chemotherapy, radiation therapy and immunotherapy agents 11, 12. However, the data on the incidence of ATRX expression and clinical significance in epithelial carcinomas is limited. It is reported that ATRX loss predicts good prognosis in cervical carcinoma and hepatocarcinoma 13, 14. And ATRX mutation results in increased immune checkpoint inhibitor (ICI) sensitivity in NSCLC 12. However, the significance of ATRX expression in the prognosis of NPC remains unknown.

In the present study, we evaluated the ATRX expression in NPC patients via immunohistochemistry and analyzed the correlation between ATRX expression and clinicopathological characteristics and patient's prognosis, and demonstrated that ATRX deficiency is correlated with earlier T stage and longer overall survival in NPC patients.

## Materials and Methods

### Patients enrollment and clinical data

Patients who were diagnosed with NPC at the Second Xiangya Hospital of Central South University (Hunan, China) between 2011 and 2016 were retrospectively reviewed. And patients without complete clinical data or unavailable paraffin blocks were excluded. Finally, a total of 227 patients were enrolled in this study. Two pathologists (HH Wang and SQ Fan) independently reviewed all H&E staining and IHC staining slides. And all the clinical and pathological characteristics were obtained from electronic medical records system of the hospital. Approval for using the patient material in this study was obtained from the Ethics Committee of the Second Xiangya Hospital of Central South University. All procedures were conducted in accordance with the declaration of Helsinki. Tissue samples were obtained from the Department of Pathology of the Second Xiangya Hospital of Central South University.

### Immunohistochemical staining and semi-quantitative scores

**Figure 1:**
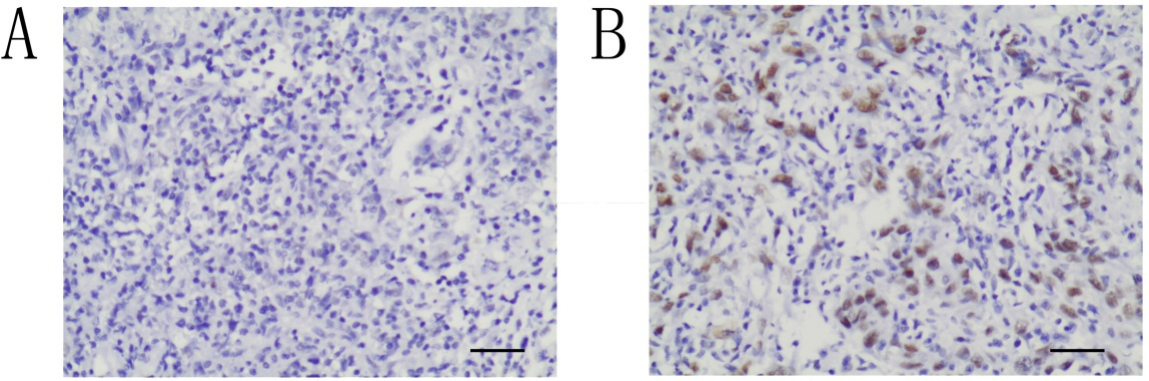
Immunohistochemical study of ATRX expression in NPC tissues. The intensity of nucleus staining was graded as negative (**A**) or positive (**B**) (A-B, ×200).

Immunohistochemical staining for ATRX was performed with 4-μm-thick sections from paraffin blocks using the MaxVision HRP polymer anti-rat IHC kit. Each section was deparaffinized and rehydrated, and high-temperature antigen retrieval was achieved for all antibodies by heating the samples in 0.01 M citrate buffer in a domestic microwave oven at full power (1000 W) for 15 mins. Besides internal positive control, positive control slides were included in every experiment. Methanol containing 3% H_2_O_2_ was applied for fifteen minutes to inactivate endogenous peroxidase. The slides were incubated with a rabbit monoclonal antibody against human, a 1:200 dilution of the primary antibody to ATRX (Sigma, Sigma-Aldrich, St. Louis, MO, USA) at 4 °C overnight. Then, all the slides were rinsed with PBS three times for 5 mins each. After the incubation with secondary antibody, the visualization signal was conducted with 3,3′-diaminobenzidine tetrachloride. Subsequently, all slides were counterstained with hematoxylin. The specificity of the antibody was determined with matched IgG isotype antibody as a negative control. Tissue samples were evaluated independently by HHW and SQF who were blinded to the clinicopathological data, at 200× magnification light microscopy. Nucleus staining of the tumor cells was assessed. Representative microscopic photos are shown in **Figure 1**. According to the proportion of nucleus staining of the tumor cells, the ATRX expression was classified as negative and positive. ATRX staining loss of tumor nucleus was defined as ATRX negative while ATRX positive was defined as that ATRX is expressed in at least 1% of nuclei in the tumor cells. The concordance rate between the two pathologist is 97%.

### Statistical analysis

Statistical analysis was performed using SPSS software V.22 (IBM, Armonk, USA). The χ² test was used to evaluate the association between ATRX expression and clinicopathological parameters including Age, Gender, T stage, N stage and clinical stage according to AJCC 8^th^ staging system. Progression-free survival (PFS) and overall survival (OS) curves were plotted using Kaplan-Meier method, and the log-rank test was used to compare the differences. P<0.05 was considered as statistically significant.

## Results

### Patient characteristics

The clinicopathological characteristics of the patients are summarized in **Table 1**. A total of 227 patients were enrolled, among which 138 (60.8%) patients were less than 50 years old, with a median age of 48 years. 156 (68.7%) patients were men, with a male-to-female ratio of 2.2:1. All patients were diagnosed with NPC and treated with chemoradiation therapy. 26 (11.5%) were T1 stage, 88 (38.8%) were T2 stage, 68 (30.0%) were T3 stage and 45 (19.8%) were T4 stage. 15 (6.6%) were N0 stage, 37 (16.3%) were N1 stage, 144 (63.4%) were N2 stage and 31 (13.7%) were N3 stage. According to the 8th AJCC staging system, 24 (10.6%) were stage II, 132 (58.1%) were stage III and 71 (31.3%) were stage IV. A total of 103 (45.4%) patients received concurrent chemoradiation, and 124 (54.6%) received sequential chemoradiation. Among all the patients, 145 (63.9%) received cisplatin-based chemotherapy, 68 (30.0%) received nedaplatin-based chemotherapy, while 14 (6.1%) received other agents.

### Correlations between ATRX expression and clinicopathological factors

NPC patients with ATRX expression, defined as ATRX positive staining in at least 1% of nuclei in the tumor cells, consist of 76.7% (174/227) of the cohort. And 23.3% (53/227) of the cohort had ATRX loss. The association between ATRX expression and the clinicopathological factors are shown in **Table 2**. ATRX loss was significantly correlated with earlier T stage (p=0.045). Statistical significance was not found in sex, age, ECOG, smoking history, hemoglobin level, treatment mode, chemotherapy agent type, N stage and AJCC stages.

### Impact of ATRX expression on the prognosis of NPC patients

**Figure 2:**
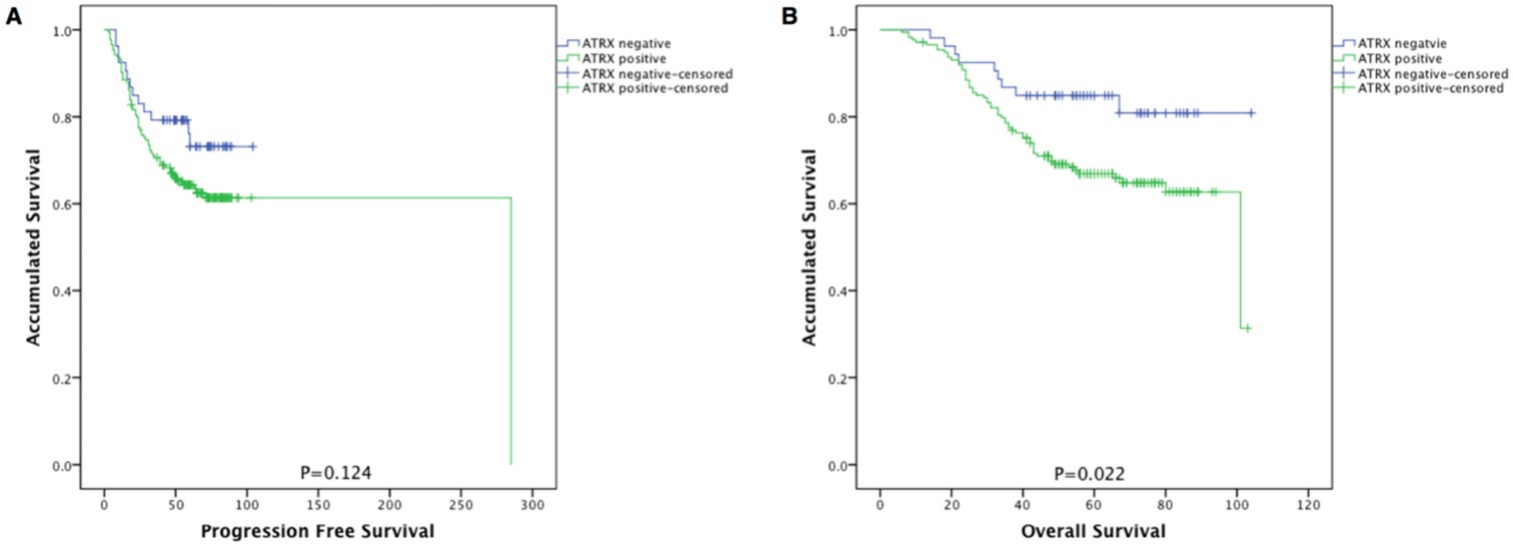
Kaplan-Meier survival curves of patients with NPC stratified by ATRX expression. (**A**) Progression-free survival and (**B**) overall survival according to ATRX expression in NPC patients. ATRX negative (n=53) and ATRX positive (n=174).

As shown in **Figure 2**, NPC patients with ATRX loss had significantly longer overall survival than those with ATRX expression (84.9% vs 66.9%, P=0.022). No prognostic significance between ATRX expression and progression free survival (PFS) was observed (73.1% vs 65.9%, p=0.124).

## Discussion

In the present study, we investigated the expression of ATRX in NPC and its correlation with clinicopathological characteristics and prognosis of NPC patients. ATRX protein expression was absent in 24.2% of NPC patients, and ATRX loss is correlated with earlier T stage and longer OS in NPC patients.

ATRX is a member of the SWI/SNF chromatin remodeling superfamily which is involved in remodeling and stabilizing genome 15. It is reported that SWI/SNF genes are found to be mutated in almost 25% of malignancies, which have prognostic significance 16. Moreover, expression changes of specific SWI/SNF chromatin remodeling proteins are also implicated as prognostic biomarkers 16. However, the consequences of mutations or expression alterations on prognoses are highly context-specific 16. ATRX mutation is mostly seen in mesenchymal malignances, including glioma, sarcoma, and neuroendocrine tumors, while the report on the incidence and prognostic significance on epithelial malignancies is rare. Loss of ATRX results in impaired genome damage repair system, which leads to genetic instability, microsatellite instability (MSI), and more aggressive phenotype 17. At the same time, it also sensitizes cancer cells to treatment agents, including DNA-damaging agents, radiotherapy 11, 18, and immune checkpoint inhibitors 12, 19. The prognostic significance of ATRX mutation on cancer patients varies between researches. It is reported that ATRX mutation is a poor prognostic factor in pancreatic neuroendocrine tumors (pNETs) 20, hepatic angiosarcomas 21, and leiomyosarcoma 22, 23, while it is found to be a good prognostic factor in glioma 24, and cervical cancer 13. Such complexities without universal good prognosis may suggest that mutations affecting chromatin remodeling genes or expression altering chromatin remodeling proteins do not directly determine the aggressiveness or prognosis of malignancies 16. Besides, this discrepancy may be resulted from patient selection and treatment modality. In the present study, we firstly reported the incidence of ATRX deficiency in NPC patients, and found that ATRX deficiency predicts good prognosis in NPC patients. However, the underlying mechanism is not fully understood, and it may be related with the following aspects. Firstly, since the ATRX protein plays significant epigenetic roles in maintaining telomere length via depositing histones at heterochromatin and telomeric DNA, tumors with ATRX mutations or loss exhibit altered telomeres and telomere dysfunction 25, 26. Moreover, ATRX mutations increase tumor mutational burden (TMB), PD-L1 expression, as well as manifested interferon gamma (IFN-γ) signaling 12, 19. Given that radiotherapy or chemoradiotherapy is the primary treatment for NPC, and the potential role of ATRX mutation in glioma patients treated with RT has been reported. Collectively, the mechanism of ATRX mutation conferring survival advantages in NPC is worth further investigation.

Mutations in ATRX are thought to result in loss of ATRX protein which mediates loss of function (LOF), and therefore in the present study, immunohistochemistry (IHC) is used as a surrogate to detect mutations. In previous research in glioma, pancreatic neuroendocrine tumors, and neuroblastoma have demonstrated that ATRX mutations are associated with absence of ATRX protein. However, in another research, Chami et al. 27 reported that using IHC as a surrogate may miss ATRX mutations in neuroblastoma, which indicated detecting ATRX mutation by IHC protein expression as a surrogate still needs further validation. Furthermore, the consistency between ATRX protein absence and ATRX mutation in NPC is worth further investigation.

Although this study identified the prognostic significance of ATRX loss in NPCs, there are several limitations. First, confirmation of ATRX mutations by other techniques, such as sequencing from fresh tissue was not included. Additionally, this study is a retrospective study, and due to a limited sample size, there is a need for validation in larger cohorts with clinical, molecular alterations, conjointly with protein expression/activation.

In conclusion, we evaluated the clinicopathological significance of ATRX expression in 227 patients with NPC. ATRX loss was significantly associated with good prognosis in patients.

## Figures and Tables

**Table 1:** Baseline characteristics of patients with NPC

Clinicopathological characteristics	Number (%)
**Age**	
≤50	138 (60.8%)
>50	89 (39.2%)
**Gender**	
Male	156 (68.7%)
Female	71 (31.3%)
**ECOG**	
0	186 (81.9%)
1	41 (18.1%)
**Smoking history**	
No	93 (40.9%)
Yes	134 (59.1%)
**HGB (M/F) (g/L)**	
<120/110	9 (4.0%)
≥120/110	218 (96.0%)
**Treatment mode**	
Concurrent chemoradiation	103 (45.4%)
Sequential chemoradiation	124 (54.6%)
**Chemotherapy agent type**	
Cisplatin	145 (63.9%)
Nedaplatin	68 (30.0%)
Others	14 (6.1%)
**Total platin dose (mg/m^2^)**	
≥300	164 (72.2%)
<300	63 (27.8%)
**ECOG**	
0	185 (81.5%)
1	42 (18.5%)
**Smoking history**	
No	93 (41.0%)
Yes	134 (59.0%)
**HGB (M/F) (g/L)**	
<120/110	15(6.6%)
≥120/110	212 (93.4%)
**Treatment mode**	
Concurrent chemoradiation	103 (45.4%)
Sequential chemoradiation	124 (54.6%)
**Chemotherapy agent type**	
Cisplatin	145 (63.9%)
Nedaplatin	68 (30.0%)
Others	14 (6.1%)
**T stages**	
T1	26 (11.5%)
T2	88 (38.8%)
T3	68 (30.0%)
T4	45 (19.8%)
**N stages**	
N0	15 (6.6%)
N1	37 (16.3%)
N2	144 (63.4%)
N3	31 (13.7%)
**AJCC stages**	
II	24 (10.6%)
III	132 (58.1%)
IV	71 (31.3%)

**Table 2:** Correlation between ATRX expression and clinicopathological factors in NPC

Clinicopathological factors	N	ATRX expression		
		negative	positive	P value
**Gender**				0.630
Male	156	35 (66.0%)	121 (69.5%)	
Female	71	18 (34.0%)	53 (30.5%)	
**Age**				
≤50	138	28 (52.8%)	110 (63.2%)	0.175
>50	89	25 (47.2%)	64 (36.4%)	
**ECOG**				0.559
0	186	46 (84.9%)	140 (81.4%)	
1	41	9 (15.1%)	32 (18.6%)	
**Smoking history**				0.671
No	93	24 (43.4%)	69 (40.1%)	
Yes	134	31 (56.6%)	103 (59.9%)	
**HGB (M/F) (g/L)**				0.370
<120/110	9	1 (1.9%)	8 (4.7%)	
≥120/110	218	54 (98.1%)	164 (95.3%)	
**Treatment mode**				0.759
Concurrent chemoradiation	103	26 (47.2%)	77 (44.8%)	
Sequential chemoradiation	124	29 (52.8%)	95 (55.2%)	
**Chemotherapy agent type**				0.291
Cisplatin	145	32 (58.5%)	113 (65.7%)	
Nedaplatin	68	21 (37.7%)	47 (27.3%)	
Others	14	2 (3.8%)	12 (7.0%)	
**T stages**				0.045
T1-2	114	33 (62.3%)	81 (46.6%)	
T3-4	113	20 (37.7%)	93 (53.4%)	
**N stages**				0.670
N0-N1	52	11 (20.8%)	41 (23.6%)	
N2-N3	175	42 (79.2%)	133 (76.4%)	
**AJCC stages**				0.383
II-III	156	39 (73.6%)	117 (67.2%)	
IV	71	14 (26.4%)	57 (32.8%)	

## Abbreviations

NPC: nasopharyngeal carcinoma
ATRX: alpha thalassemia/mental retardation X-linked
SWI/SNF: switch/sucrose nonfermentable
ICI: immune checkpoint inhibitor
PFS: progression-free survival
OS: overall survival
MSI: microsatellite instability
pNETS: pancreatic neuroendocrine tumors
TMB: tumor mutational burden
IFN-γ: interferon gamma
LOF: loss of function
IHC: immunohistochemistry
